# Unwarranted variations in end-of-life care and the impact of using an electronic coordination system

**DOI:** 10.1007/s43999-023-00019-5

**Published:** 2023-03-08

**Authors:** Karen Chumbley, Tim Wilson, Erica Ison, Andi Orlowski

**Affiliations:** 1 St Helena Hospice, Barncroft Close, Colchester, CO49JU UK; 2 Oxford Centre for Triple Value Healthcare Ltd, Summertown Pavilion, Middle Way, Oxford, OX2 7LG UK; 3 Department of Primary Care and Public Health, Imperial College London, London, UK

**Keywords:** End of life, Palliative care, Unwarranted variation, Equity, Electronic records

## Abstract

This study looks at the variations in end-of-life care in North-East Essex (eastern England) combining hospital records, official death records and the local electronic end-of-life coordination tool. These differences included dying in hospital (versus a general wish to die in the usual place of residence), and inequity in care provision: the place of death varying according to the cause of death (even for highly predictable conditions); and deprivation being associated with a greater likelihood of dying in hospital. There was a positive correlation between the use of an electronic end-of-life coordination system and dying in the preferred place of care. The results suggest two actions for policy makers. First, look at variations in end-of-life care so that areas of need can be identified. Second, use of an electronic end-of-life coordination tool is correlated with a reduction in unwarranted variation in the place of death.

## Introduction

A core tenet of the English NHS, and other universal healthcare systems, is the equitable provision of high value and high-quality healthcare and minimisation of healthcare amenable inequalities. This is enshrined in the legal duty of everyone in the NHS to consider, in their decision making, three aims:

1. The impact on the health and well-being of the populations served, including inequalities in those outcomes;
2. The impact on access to high quality healthcare services, including inequalities in access; and
3. Using NHS resources sustainably and efficiently [1].

However, usual approaches to data reporting do not really give a good view of performance against these three aims, including the impact of variation on the achievement of the aims.

A population approach to end-of-life care focuses on the needs of the whole population, with the aims of decreasing inequity and improving outcomes that matter to the people served with the resources available (value) [2]. For instance, most people at the end of life prefer to be cared for at home [3]. It is considered good clinical practice for every individual to have an opportunity to consider and share their preferences for care at the end of life [4, 5]. Further, a recent systematic review of 40 high quality studies showed inequities in the delivery of care for people at the end of life [6].

In England it is recommended that every person who wants to do so should be able to record their preferences for end-of-life care, and for those preferences to be held on an electronic palliative care co-ordination system (EPaCCS) [7]. These systems are secure databases that allow relevant health and care professionals to access a person’s preferences and where possible coordinate their care accordingly. In some studies, the use of an EPaCCS has been found to be associated with a lower rate of emergency admission to hospital [8]. A systematic review of EPaCCS use, however, found a paucity of good-quality evidence about their effect on patient outcomes, and the authors recommended further research [9].

In 2013 an EPaCCS was commissioned in North East Essex (NEE), locally known as the My Care Choices Register, to serve a population of about 350,000. In a local evaluation, a high achievement of preferred place of care was demonstrated for people who had recorded their choices, and only 3.5% of people recorded a preference for end-of-life care in hospital [10].

As a group with oversight of the resources used for people at the end of life in North East Essex, we wanted to be able to make informed decisions about how to better deliver services to achieve the three aims. We did not set out to do a research study, but instead use research methods such as variation, differences and correlation as a means to identify value improvement opportunities. It was our belief that by looking at variation, and social, clinical, or administrative differences between population groups we would be able to have a better assessment of the achievement of the three aims in relation to people at the end of life. We further hypothesized that people registered with general practices that had high levels of EPaCCS use would be more likely to die out of hospital when compared with people registered with general practices that had lower levels of EPaCCS use.

## Method

People of all ages who died in North-East Essex between April 2018 and March 2019 were identified from a linked Hospital Episode Statistics (HES) and Office of National Statistics (ONS) dataset. The identity of the general practice at which the person was registered was identified. The ONS definition of cause of death was used to determine whether each person died from cancer or from other causes. The deprivation status of the area of residency of the person that died was identified by using the first elements of their postcodes and linking this to the national database of indices of multiple deprivation [11].

Variations between general practices across North East Essex were analysed, as were differences in the cause of death (cancer and non-cancer deaths) and the degree of deprivation. Reporting was limited to descriptive statistics. It was not possible to conduct a multi-variate analysis.

The EPaCCS dataset was used in combination with the total number of deaths from the ONS dataset to calculate the percentage of people at each general practice in North East Essex who died with an EPaCCS record in place. This was used as an indicator of a practice’s level of EPaCCS use. General practices were assigned to high, medium and low EPaCCS usage groups based on the percentage of deaths with an EPaCCS record. Because we were analysing data to support management decisions, and this was not a research study, a pragmatic approach was taken to determining high, medium, and low EPaCCS usage. The high usage group (*n* = 7) was defined as practices with percentage deaths with an EPaCCS record > 30%; the medium usage group (*n* = 9) was defined as practices with percentage deaths with an EPaCCS record > 20% and < = 30%; the low usage group (*n* = 16) was defined as practices with percentage deaths with an EPaCCS record <=20%.

The linked HES-ONS dataset made it possible to capture the place of death by general practice and to determine the percentage of people who died in hospital as a proportion of total deaths. This was then compared with the percentage of people who had an EPaCCS in place at the time of death.

This method was followed for all people, for people who died of cancer and for people who died of non-cancer conditions. Statistical significance was determined using a one-way ANOVA in Tableau.

## Results

**Fig. 1:**
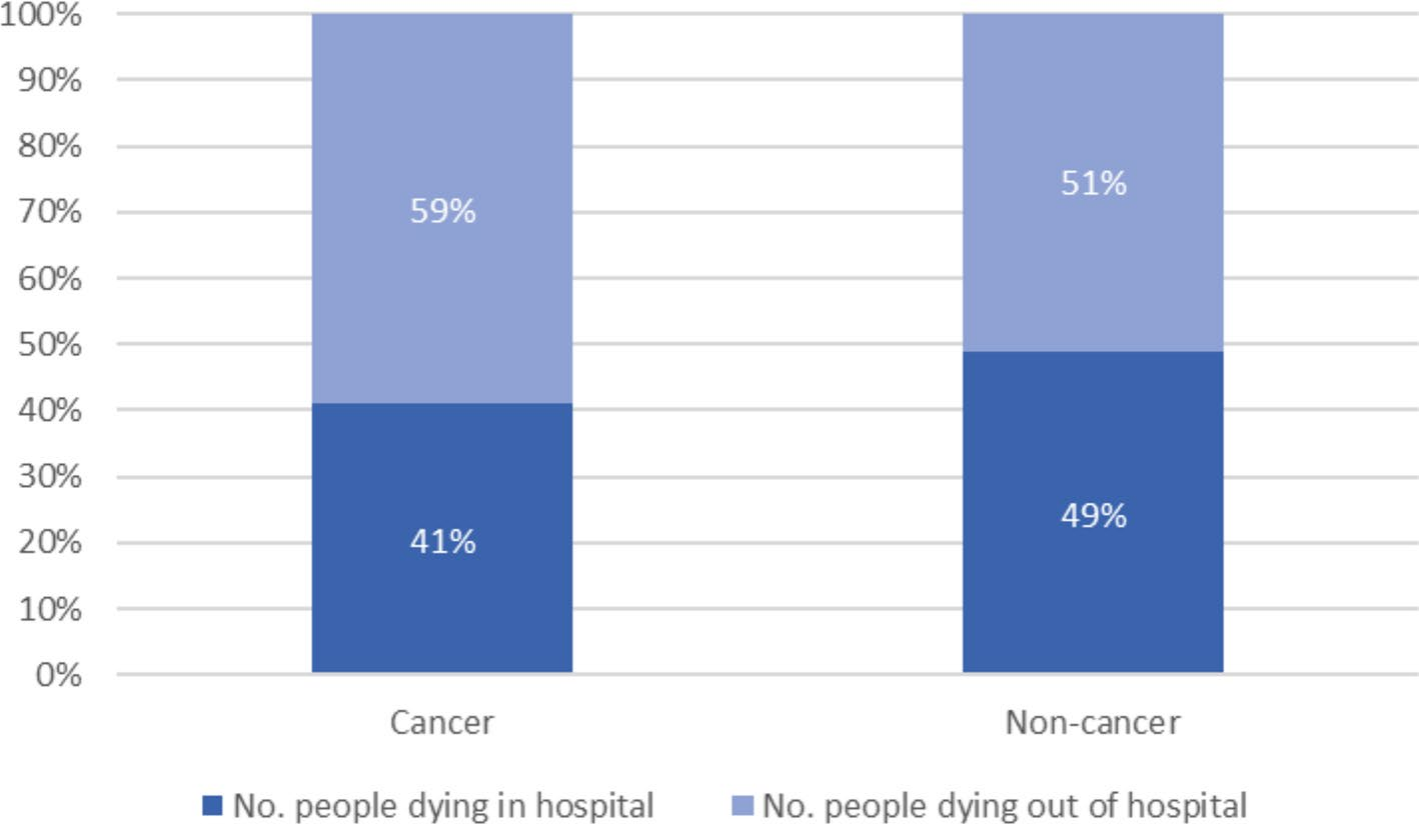
Percentage (%) of people in the last year of life in North East Essex dying from cancer and non-cancer conditions according to place of death (in hospital; out of hospital), 2018/19

There was a variation in the place of death when considering the cause of death (cancer or non-cancer). As can be seen from Fig. 1, in 2018/19, almost three-fifths (59%) of people with cancer in North East Essex died out of hospital than in (41%), whereas only slightly more than half of people with non-cancer conditions in North East Essex die out of hospital (51%) than in (49%) (see Fig. 1).

**Fig. 2:**
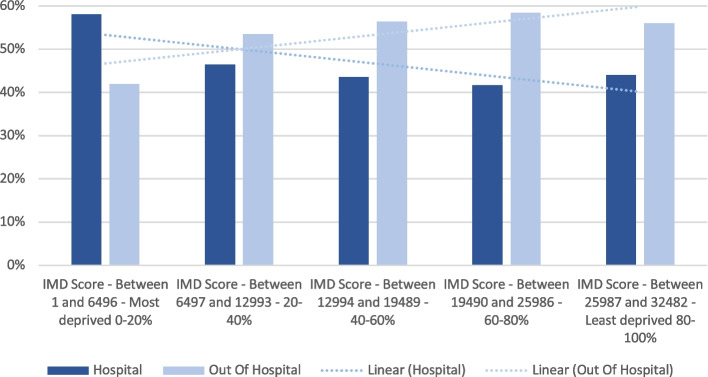
Place of death (in hospital; out of hospital) for people in the last year of life in North East Essex according to IMD score, 2018/19

When considering the quintile of indices of deprivation, there was a variation of place of death that appeared to moderately correlate with the degree of deprivation *R*^2^ = 0.63 (Pearson correlation, see Fig. 2).

**Fig. 3:**
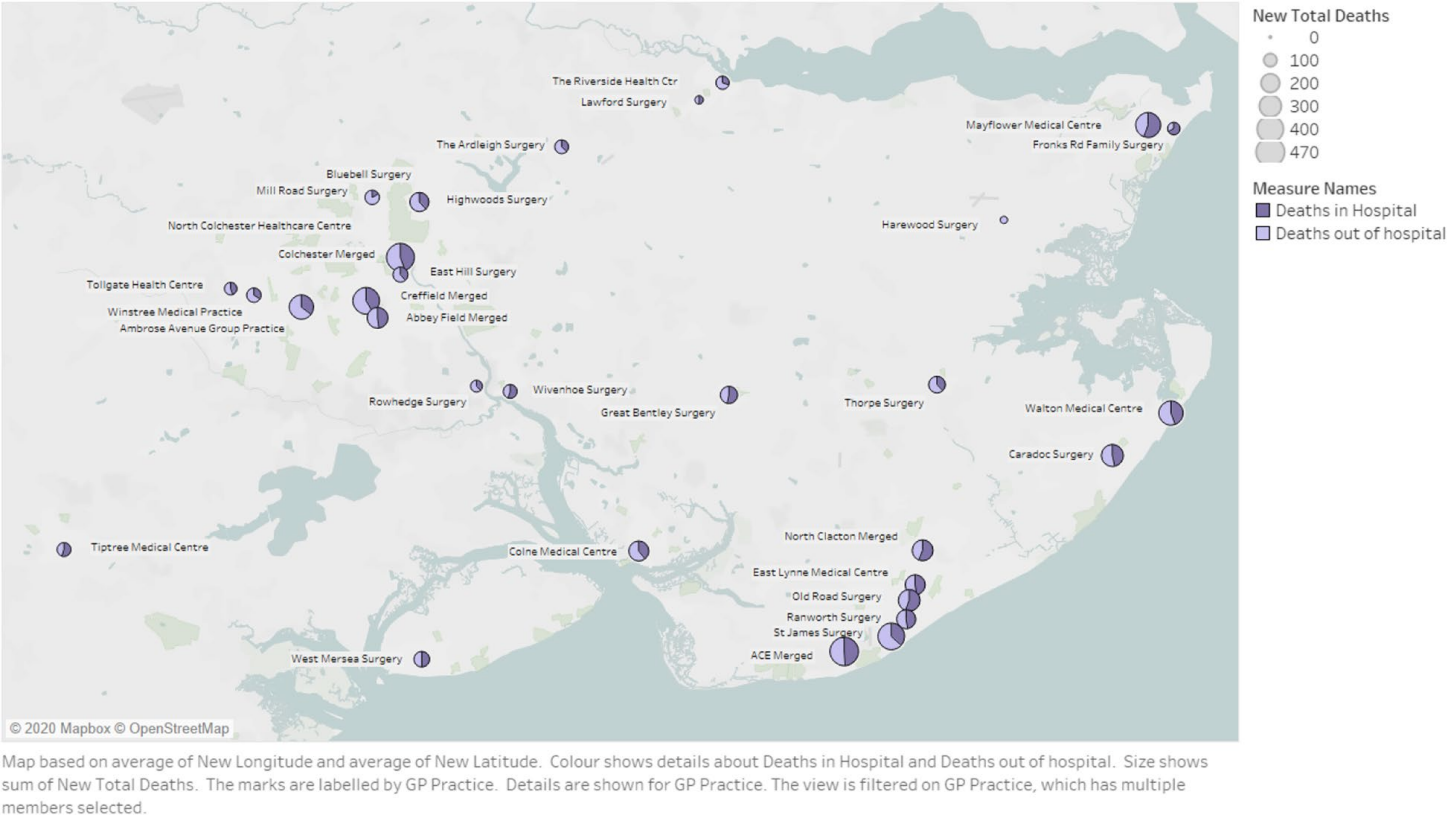
Number of deaths (in hospital; out of hospital) by general practice in North East Essex, 2018/19. (Source ICHP analysis)

In Fig. 3, the number of deaths in North East Essex has been shown for each general practice. Figure 3 shows the number of deaths across North East Essex. For each general practice, the number of deaths in hospital in relation to the number of deaths out of hospital has been visualised as a pie chart (i.e. within each circle); the size of each circle represents the number of deaths recorded. The number of deaths out of hospital varied between 39% in the lowest general practice and 100% in the highest general practice. It should be noted that many cases general practices serving the same neighbourhoods have markedly different results, although a pattern around Colchester and Clacton might be inferred. Unfortunately, the statistical significance of this could not be tested, which would be required to draw a firm conclusion.

As can be seen, there are large variations in the place of death depending on which general practice the decedent was registered with.

**Fig. 4:**
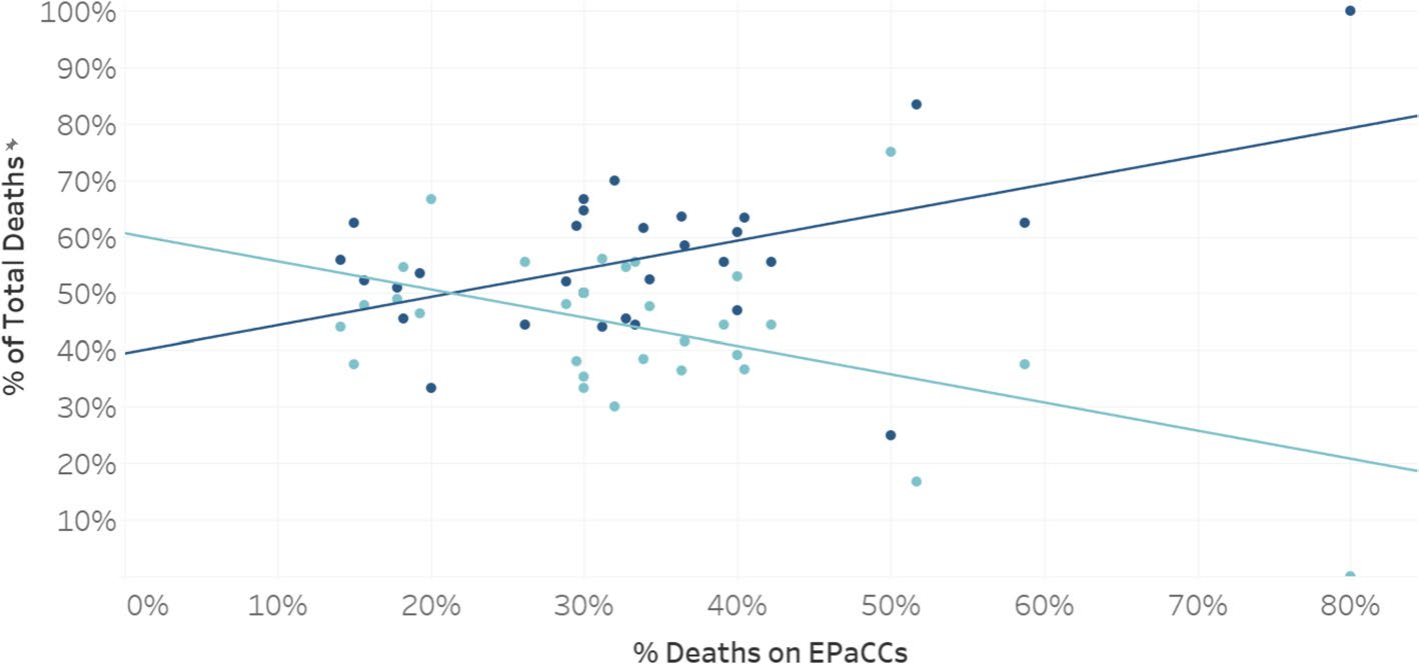
Place of death (in hospital; out of hospital) for people in the last year of life in North East Essex in relation to EPaCCS usage by the general practice at which they were registered, 2018/19. Light blue dots represent deaths in hospital; dark blue dots represent deaths out of hospital

A statistically significant (*p* < 0.01) correlation was found between higher levels of EPaCCS use by a general practice and a greater likelihood of a person registered with that practice dying out of hospital (Fig. 4).

The correlation between higher levels of EPaCCS use by a general practice and a greater likelihood of a person registered with that practice dying of out of hospital if they died of cancer was statistically significant (*p* < 0.01).

The correlation between higher levels of EPaCCS use by a general practice and a greater likelihood of a person registered with that practice dying of out of hospital if they died of a non-cancer condition was not statistically significant (*p* = 0.3); however, there was a statistically significant difference between the general practices that were the highest users of EPaCCS and those that were the lowest users of EPaCCS, that is, people who had non-cancer conditions and were registered at practices with high EPaCCS use had a greater likelihood of dying out of hospital than people with non-cancer conditions registered at practices with low EPaCCS use (*p* < 0.05).

## Discussion

### Summary

In North East Essex most people who record their end-of-life care choices, including their preferred place of care, prefer not to die in hospital. There are variations in the likelihood of dying in hospital or out of hospital that appear to be related to deprivation. In line with other studies, in North East Essex, if you are from a more deprived neighbourhood, you are more likely to die in hospital than if you are from a less deprived neighbourhood [12]. In future, we would be keen to conduct a similar equity analysis using other protected characteristics such as gender, ethnicity and alike, although this will need the necessary clinical coding which is not always in place. We would also like to look at the equitable use of EPaCCS and whether this is a driving force in the difference in outcomes.

There is a difference between people dying of cancer compared to dying of non-cancer (although this was not statistically significant). However, this difference may be due to non-cancer conditions including unexpected deaths (e.g., due to trauma, suicide, stroke or cardiac arrest- see limitations below). Nevertheless, there have been many years of focussing palliative care on people dying of cancer conditions; providing the same focus on conditions that are amenable to good end-of-life care, such as heart failure, COPD or people dying with dementia is likely to be valuable. A similar impact of cause of death on place of death has been observed in other studies [13, 14].

There are variations between general practices regarding the place of death, even between geographically adjacent general practices serving the same neighbourhood population. This suggests that the driving factors causing variation are not rurality, the size of the practice or similar. Anecdotally, knowledge of the general practices suggest it was internal characteristics, such as an enthusiastic clinician with end-of-life expertise. Improving variation would therefore need to be targeted at internal general practice factors.

A recent systematic review had not found any research showing an association between EPaCCS use and place of death [9]. Our data suggests that for all people, and for people dying of cancer and of non-cancer conditions, being registered with a general practice that has a high level of EPaCCS use is associated with a statistically significant higher probability of dying out of hospital.

This study adds to the growing body of evidence that establishing a person’s preferences for care at the end of life is likely to lead to outcomes in line with people’s preferences [15]. It adds weight to using EPaCCS as a tool in recording and sharing people’s preferences for end-of-life care. It has also allowed us to consider specific targeted support packages in areas where outcomes appear worse, as a mean to reduce inequities in care. We shall be testing these packages through continued monitoring.

### Limitations

In many cases we were unable to test for statistical significance, or no statistical significance was found (possibly due to sample size). However, this project was not primarily a research project looking for statistical significance, but an analysis of the data to guide local decision makers. We were unable to determine whether there was an association between different factors. For instance, were people in deprived areas, served by particular general practices more likely to die of non-cancerous conditions. The map suggested this was not an issue, but it cannot be ruled out.

In classifying deaths into cancer and non-cancer, the latter group would have included a number of unexpected deaths including from cardiac arrest, suicide, trauma and stroke. None of these are amenable to end-of-life care. However, future work and analysis is looking at specific non-cancer conditions such as heart failure, COPD and people dying with dementia.

The grouping of general practices into high, medium, and low EPaCCS usages groups was done pragmatically to allow for a reasonable distribution of practice numbers between groups. Different grouping might have altered the results somewhat, although they would not have changed the general, non-grouped, correlation seen in Fig. 4.

One limitation of this study is that we were not able to link the EPaCCS dataset with the linked HES-ONS dataset, which would have allowed investigation at the individual level rather than at the level of general practices.

General practices with high levels of EPaCCS use might have shared characteristics, resulting in a greater likelihood of people at end of life dying out of hospital in addition to the routine use of an EPaCCS. Although the evidence for advance care planning is compelling, there are other factors that could influence higher out-of-hospital death rates such as the presence at a general practice of a clinician with an interest in end-of-life care. Practices with low EPaCCS use may also have shared characteristics, such as poor continuity of care or high vacancy rates.

### Implications for practice

Variations in the outcomes experienced by people from more deprived neighbourhoods are iniquitous and need to be addressed. We are targeting specific support packages to more deprived neighbourhoods because of this project. Variations in the outcomes experienced by people dying of cancerous and non-cancerous conditions need to be mitigated by better planning and awareness of palliative care for non-cancerous conditions. We already have a programme in place for people with dementia and will be extending it to other conditions because of this project.

Variations in general practice end of life care should be mitigated through support and training. We are testing a programme of targeted support and training because of this project.

Investment in the sharing of advance care plans through digital systems can support people to be cared for in the community.

## Data Availability

The sources of the data and material is described in the paper. It is a mix of publicly available data and fully anonymised data from the local hospice.
